# Minimal Detectable Change and Minimal Clinically Important Difference of Bruininks-Oseretsky Test of Motor Proficiency Second Edition Balance Component in Patients With Cerebral Palsy: A Pilot Study

**DOI:** 10.7759/cureus.110434

**Published:** 2026-06-08

**Authors:** Abhijit N Satralkar, Suvarna Ganvir, Sanjeev Kumar

**Affiliations:** 1 Department of Neurophysiotherapy, AIMS College of Physiotherapy, Dombivli, IND; 2 Department of Neurophysiotherapy, Dr. Vithalrao Vikhe Patil Foundation's College of Physiotherapy, Ahilyanagar, IND; 3 Department of Physiotherapy, KLE Institute of Physiotherapy, Belagavi, IND

**Keywords:** cerebral palsy, minimal clinically important difference, physical therapy modalities, postural balance, psychometrics

## Abstract

Background

Balance deficits are among the most disabling features of spastic cerebral palsy (CP), limiting mobility and participation. The balance subtest of the Bruininks-Oseretsky Test of Motor Proficiency Second Edition (BOT-2) in paediatric physiotherapy is widely used; however, its minimal clinically important difference (MCID) and minimal detectable change (MDC) for spastic CP children have not been reported. Hence, in spastic CP children, the current study primarily aimed to determine the MCID and MDC for the balance subtest of the BOT-2 and, as a secondary objective, to examine its concurrent validity with the Pediatric Balance Scale (PBS).

Methods

A total of 30 spastic CP children (Gross Motor Function Classification System (GMFCS) Levels I-II; mean age 8.35 ± 1.93 years) were assessed using the PBS and balance subtest of BOT-2 by two independent physiotherapists at baseline and after four weeks of conventional physiotherapy. The MDC and MCID were estimated with the PBS as the external anchor. Paired t-tests and Pearson correlation coefficients were applied for inferential analysis.

Results

Statistically significant improvements were observed in BOT-2 Balance and PBS scores for both assessors (p < 0.001). The MDC ranged from 1.5 to 1.7 points. Moderate-to-strong correlations were identified between BOT-2 and PBS change scores (r = 0.583-0.604, p ≤0.001). An MCID of ≥3.5 points across both assessors was consistently identified, with good-to-excellent discriminative capacity (AUC = 0.814-0.884).

Conclusion

The BOT-2 Balance subtest demonstrates good responsiveness in children with spastic CP. These preliminary findings provide an MDC of 1.5-1.7 points and an MCID of ≥3.5 points, providing evidence-based benchmarks for interpreting clinically meaningful balance improvement, supporting the integration of BOT-2 Balance into routine paediatric physiotherapy assessment.

## Introduction

In childhood, cerebral palsy (CP), which affects approximately 2-3.5 per 1,000 live births worldwide, is the most common cause of physical disability [1,2]. It occurs during the development of the foetal or infant brain, which involves permanent disorders of movement and posture attributable to non-progressive disturbances [3,4], which include sensation, communication, cognition, perception, and behaviour, and secondary musculoskeletal complications, leading to diminished quality of life and reduced functional independence [3,5].

Balance deficits associated with CP are particularly disabling. Difficulties in sensory integration, reduced selective motor control, and impaired static and dynamic postural control owing to abnormal muscle tone are commonly observed in children with spastic CP [5-8]. In daily life, limited participation in age-appropriate activities and mobility restrictions are commonly observed due to balance impairments, which hinder independence [5,9-11]. Furthermore, an additional burden is imposed on the child and caregivers, as the risk of falls and injury increases due to impaired balance. Therefore, critical components of both clinical practice and rehabilitation research involve accurate assessment of balance and the ability to detect and interpret meaningful change over time [8,12].

In children aged 4-21 years, a widely used standardised, norm-referenced tool designed to assess motor skills, both fine and gross, is the Bruininks-Oseretsky Test of Motor Proficiency, Second Edition (BOT-2) [2,13,14]. Static and dynamic postural control are challenged through the balance component of BOT-2, as it consists of a range of tasks. In paediatric physiotherapy research and clinical practice, the BOT-2 is increasingly being employed as an outcome measure as it has demonstrated acceptable validity and reliability [15-19]. However, despite its widespread use in CP children, evidence regarding the BOT-2 Balance subtest clinimetric properties, Minimal Detectable Change (MDC), and the Minimal Clinically Important Difference (MCID) remains sparse [15-19].

MDC reflects the smallest amount of change in a score that exceeds the inherent measurement error of an instrument, indicating a statistically reliable change rather than random fluctuation [8,20,21]. In contrast, MCID represents the smallest change in an outcome that is perceived as beneficial or meaningful by the patient, caregiver, or clinician and would warrant a change in patient management [9,22,23]. While MDC is a statistical property based on measurement reliability, MCID is an anchor-based indicator of clinical relevance. Without established MDC and MCID values, clinicians cannot confidently distinguish true therapeutic gains from measurement noise or determine whether observed changes are practically meaningful [7,23,24]. For the BOT-2 Balance component, establishing these thresholds in CP children is therefore essential to guide evidence-based clinical decision-making and outcome interpretation.

For school-age children, a 14-item functional balance assessment tool is the Pediatric Balance Scale (PBS) [20,25,26]. In CP children, strong psychometric properties (good reliability and concurrent validity) were reported, rendering it a suitable external anchor for MCID estimation [7,20,25,26]. Employing the PBS as the reference criterion, the use of an anchor-based approach provides a clinically grounded method for determining the threshold of beneficial change on the BOT-2 Balance component.

In paediatric rehabilitation, despite the increasing application of the balance subtest of BOT-2 and its clinical relevance, no previous study has established its MDC or MCID specifically for spastic CP children. In the literature regarding the measurement of the outcome, the absence of such data represents a significant gap. Accordingly, the current pilot study was executed with the primary aim of determining the MDC and MCID for the BOT-2 Balance component in spastic CP children and to explore the secondary objective, which consisted of the correlation between changes in BOT-2 Balance and PBS scores as a measure of concurrent validity.

## Materials and methods

Across tertiary care hospital clinics, physiotherapy colleges, and outpatient departments (OPDs) in and around the city, a prospective observational pilot study was conducted after approval from the Institutional Ethical Committee of Dr. Vithalrao Vikhe Patil Foundation’s College of Physiotherapy, Ahilyanagar, India, with reference number IEC/COPT/2021/PhD-07/A-25/10/2021 from November 2021 to December 2024. The purpose and methodology were explained to all the children and their parents or guardians, and written informed consent was obtained in their best-understood language prior to enrolment. Participant confidentiality was maintained throughout the study.

As a pilot study, no formal hypothesis-driven sample size calculation was performed. However, for planning purposes, an initial target sample size of 81 was estimated using OpenEpi software (version 3.01; Centers for Disease Control and Prevention, Atlanta, GA, USA) based on a finite population of 100,000, an anticipated frequency (proportion) of 5%, a 95% confidence level, a design effect of 1.0, and an absolute precision of 5%. The formula used for sample size estimation for a proportion in a finite population is n = [N × Z² × p × (1-p)] / [d² × (N-1) + Z² × p × (1-p)], where N = population size (100,000), Z = Z-score for 95% confidence (1.96), p = anticipated proportion (0.05), and d = margin of error/precision (0.05). This yielded a base sample of approximately 73, with an additional 10% (eight participants) added to account for potential dropouts. In practice, due to the study's pilot nature and recruitment constraints, a convenience sample of 30 participants who met the eligibility criteria and completed the study was enrolled. Convenience sampling was adopted due to the pilot nature of the study and practical constraints, including limited resources and time constraints. This approach enabled efficient recruitment from accessible tertiary care hospital clinics, physiotherapy colleges, and outpatient departments in and around the city. To minimise potential selection bias, all eligible children presenting to the participating centres during the study period were approached consecutively until the target sample was reached. Strict inclusion and exclusion criteria were applied to ensure a relatively homogeneous group of higher-functioning children.

The study population involved spastic CP children (aged 4-12 years, both females and males), with level I-II of the Gross Motor Function Classification System (GMFCS) [4,27], spasticity grade 1 to 1+ on the Modified Ashworth Scale (MAS), who were able to stand for at least one minute with or without support and could understand and execute assessor commands. Children with GMFCS levels III to V, who had received any treatment in the previous six months specifically for balance deficits, those with major co-morbid conditions such as moderate-to-severe intellectual disability, profound visual or hearing impairment, or significant behavioural problems, children falling in the average, below average, or well below average descriptive categories on BOT-2 [2,13,14], those in the obese range, any major trauma or fractures within the past two years, metabolic disorders, genetic disorders, progressive neurological disorders, or severe concurrent illnesses (e.g., traumatic brain injury or active pneumonia) not typically associated with CP were excluded [6,28]. Participation was entirely voluntary, and subjects were free to withdraw at any time for any reason without loss of benefits or penalty.

The thought process for data selection (participant enrolment) was driven by the need to recruit a relatively homogeneous group of higher-functioning children (GMFCS I-II) to minimise variability in a small pilot sample, while ensuring participants could safely and reliably perform the BOT-2 and PBS [20,25,26] assessments. This approach prioritised feasibility, ethical considerations (minimising the burden on more severely affected children), and the ability to generate reliable preliminary clinimetric data. All eligible children presenting to the participating centres during the study period were approached consecutively until the target feasible sample was reached.

Enrolled children continued their routine conventional physiotherapy (usual care) for a minimum of four weeks at their respective treatment centres. This included standard physiotherapy interventions such as balance training, strengthening, stretching, and functional mobility exercises, though the specific components, frequency, intensity, and duration were not standardised across participants and reflected real-world clinical variability. A follow-up was conducted weekly to ensure that all children were receiving regular conventional physiotherapy. All participants were assessed as a single group. At baseline (start of the study), balance was evaluated independently by two trained physiotherapists using both the PBS and the balance component of BOT-2 [2,13,14]. The same two-assessor protocol was repeated after a four-week interval, with assessors blinded to each other’s scores to minimise inter-rater bias. The assessors were trained prior to data collection to ensure the standardised administration of both tools.

**Figure 1:**
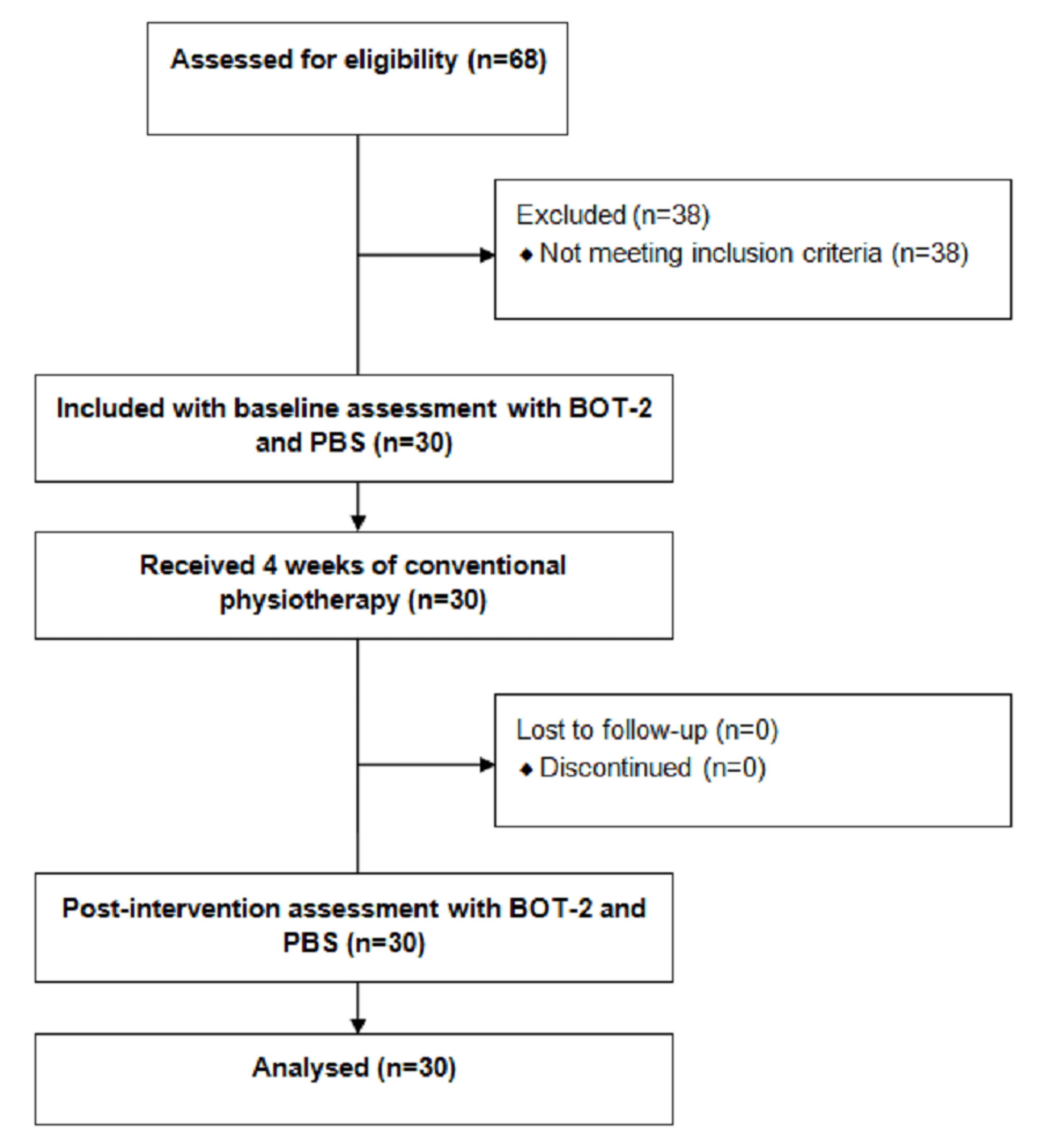
Participant flow diagram showing recruitment, exclusion, and completion of the study A total of 30 participants completed the study and were included in the final analysis. The planned sample size was 81 for pilot estimation purposes; the actual number reflects practical recruitment constraints and the pilot design. The figure was created using Microsoft Word (Microsoft Corporation, Redmond, WA, USA).

The outcome measures assessed at baseline and post-intervention (after four weeks) were the BOT-2 Balance subtest, utilised for children aged 4-21 years, which is a standardised, norm-referenced assessment of static and dynamic postural control [2,13,14]. The balance subtest comprises tasks requiring varying levels of postural challenge. Scores are reported as scaled scores. The other measure was PBS, which is a 14-item functional balance assessment for school-age children [20,25,26]. The PBS (total score of 0-56) was used as the external anchor, with higher scores indicating better balance for the MCID estimation. The participant flow diagram showing recruitment, exclusion, and completion of the study is illustrated in Figure 1.

Statistical analysis

Data were collected on demographic details, BOT-2 Balance subtest scores, and PBS scores using a standardised assessment form and were entered into Microsoft Excel (Microsoft Corporation, Redmond, Washington). To conduct statistical analysis, IBM SPSS Statistics for Windows, Version 23 (Released 2015; IBM Corp., Armonk, New York), was used. The Standard Error of Measurement (SEM) was used to calculate MDC from the intraclass correlation coefficient (ICC) of baseline scores [8,21]. MCID was estimated by using an anchor-based receiver operating characteristic (ROC) curve analysis, with the PBS score serving as the external clinical anchor [7,24]. A change in PBS was used to classify participants as 'improved' or 'not improved', and the optimal BOT-2 cut-off was identified by maximising the area under the ROC curve (AUC). The change in the PBS total score was used as the external clinical anchor. Participants were classified as 'improved' if they demonstrated a change in PBS score of ≥ 4 points, and as 'not improved' if the change was < 4 points. This threshold was selected a priori based on the distribution-based MCID (3.66 points) and anchor-based MCID (5.83 points) reported for the PBS total score in children with CP, representing a practical value within the clinically meaningful range [29,30]. To examine the relationship between changes in the PBS scores and BOT-2 Balance component, Pearson correlation coefficients were computed [17,25]. Within the group, to compare pre- and post-intervention BOT-2 and PBS scores, paired t-tests were conducted [29]. Results with p-values < 0.05 and 95% confidence intervals were considered statistically significant.

## Results

A total of 30 spastic CP children (mean age: 8.35 ± 1.93 years; range: 5-12 years) completed the study. Table 1 reports the demographic parameters.

**Table 1:** Demographic parameters GMFCS = Gross Motor Function Classification System

Parameters		n (%)
Gender	Male	17 (56.7)
	Female	13 (43.3)
Preferred drawing hand	Right	27 (90)
	Left	3 (10)
Preferred throwing hand	Right	27 (90)
	Left	3 (10)
Preferred leg/foot	Right	27 (90)
	Left	3 (10)
GMFCS level	1	28 (93.3)
	2	2 (6.7)
	Total	30 (100)

Descriptive statistics for the pre- and post-intervention balance subtest of the BOT-2 and PBS scores for both assessors, along with mean differences and MDC values, are presented in Table 2. For Assessor 1, mean pre-intervention BOT-2 Balance scores were improved post-intervention. For Assessor 2, pre-intervention BOT-2 scores were also improved post-intervention, with a mean difference and an MDC of PBS scores similarly improved. These findings indicate that the observed improvements in both BOT-2 and PBS scores substantially exceeded the MDC thresholds, affirming their clinical significance.

**Table 2:** Descriptive statistics for pre- and post-intervention balance subtest of BOT-2 and PBS scores All values are presented as mean ± SD except where otherwise specified. MDC values were derived from the Standard Error of Measurement using baseline intraclass correlation coefficients. PBS = Pediatric Balance Scale, BOT-2 = Bruininks–Oseretsky Test of Motor Proficiency Second Edition, A1 = Assessor 1, A2 = Assessor 2, N = Total number of patients, MDC = Minimal Detectable Change

Parameters	N	Minimum	Maximum	Mean	Standard Deviation	Standard Error of Mean	MDC
Pre-Treatment Assessor 01 - BOT-2 Balance Score	30	10	22	14.9	3.4	0.6	1.7
Pre-Treatment Assessor 02 - BOT-2 Balance Score	30	12	21	15.3	3.0	0.6	1.5
Pre-Treatment Assessor 01- PBS Score	30	13	44	21.2	8.9	1.6	4.5
Pre-Treatment Assessor 02 - PBS Score	30	13	44	21.5	8.9	1.6	4.5
Post-Treatment Assessor 01 - BOT-2 Balance Score	30	14	26	19.1	3.0	0.5	1.5
Post-Treatment Assessor 02 - BOT-2 Balance Score	30	13	26	19.1	3.0	0.5	1.5
Post-Treatment Assessor 01 - PBS Score	30	18	48	26.5	8.0	1.4	4.0
Post-Treatment Assessor 02 - PBS Score	30	17	50	26.8	8.5	1.5	4.3
Mean Difference BOT A1	30	1	12	4.1	2.1	0.3	1.1
Mean Difference BOT A2	30	-1	8	3.7	2.1	0.4	1.1
Mean Difference PBS A1	30	0	11	5.3	2.7	0.5	1.4
Mean Difference PBS A2	30	0	10	5.2	2.5	0.4	1.2

Paired t-test analyses revealed statistically significant improvements from pre- to post-intervention across all four assessments, as summarised in Table 3. The consistency of significant improvements across both assessors and both scales strengthens confidence in the reliability of the findings [2].

**Table 3:** Comparison of balance subtest of BOT-2 and PBS scores A paired t-test was used to compare pre- and post-intervention scores. Exact p-values were Assessor 01 - BOT-2 Balance score (pre and post) = 2.16×10⁻¹¹, Assessor 02 - BOT-2 Balance score (pre and post) = 2.64×10⁻¹⁰, Assessor 01 - PBS score (pre and post) = 2.69×10⁻¹¹, and Assessor 02 - BOT-2 Balance score (pre and post) = 3.85×10⁻¹². Data are presented as mean ± standard deviation. PBS = Pediatric Balance Scale, BOT-2 = Bruininks–Oseretsky Test of Motor Proficiency Second Edition, N = Total number of patients

Parameters		N	Mean	Standard Deviation	Standard Error Mean	Mean Difference	95% CI of Difference	Paired t-test Value	P-value
Assessor 01 - BOT-2 Balance score	Pre	30	14.9	3.39	.62	-4.1	3.3–4.9	-10.5	<0.0001
	Post	30	19.1	3.0	.54				
Assessor 02 - BOT-2 Balance score	Pre	30	15.3	3.0	.55	-3.7	2.9–4.5	-9.4	<0.0001
	Post	30	19.1	3.0	.56				
Assessor 01 - PBS score	Pre	30	21.2	8.9	1.6	-5.3	4.3–6.4	-10.4	<0.0001
	Post	30	26.5	8.0	1.4				
Assessor 02 - PBS score	Pre	30	21.5	8.9	1.6	-5.2	4.2–6.1	-11.3	<0.0001
	Post	30	26.8	8.5	1.5				

**Figure 2:**
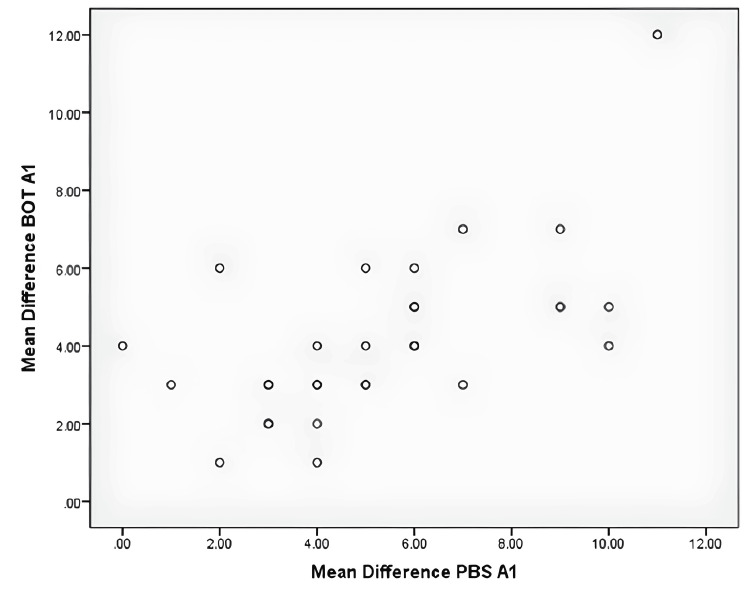
Scatter plot showing the correlation between the mean change in BOT-2 Balance subtest scores and mean change in PBS scores for Assessor 1 (r = 0.604, p < 0.001) PBS = Pediatric Balance Scale, BOT-2 = Bruininks–Oseretsky Test of Motor Proficiency Second Edition, A1 = Assessor 1 The figure was created using IBM SPSS Statistics for Windows, Version 23 (Released 2015; IBM Corp., Armonk, New York).

**Figure 3:**
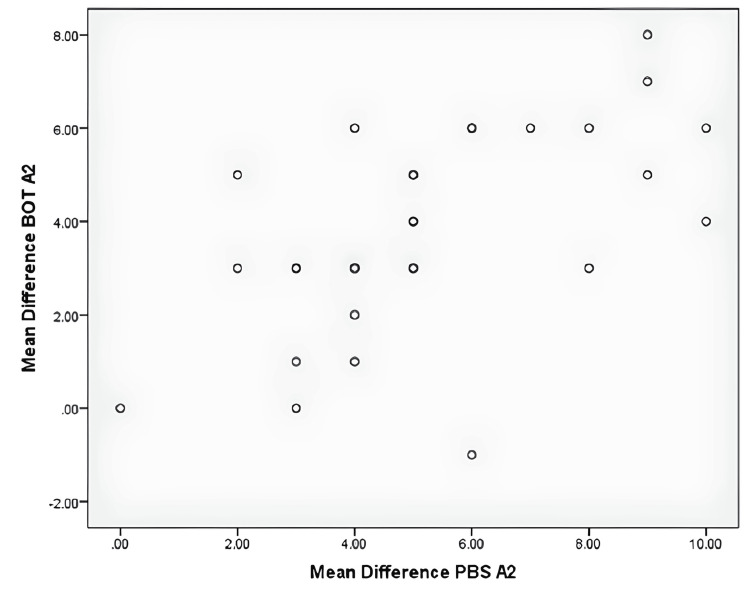
Scatter plot showing the correlation between the mean change in BOT-2 Balance subtest scores and mean change in PBS scores for Assessor 2 (r = 0.583, p = 0.001) PBS = Pediatric Balance Scale, BOT-2 = Bruininks–Oseretsky Test of Motor Proficiency Second Edition, A1 = Assessor 1 The figure was created using IBM SPSS Statistics for Windows, Version 23 (Released 2015; IBM Corp., Armonk, New York).

Pearson correlation analysis demonstrated moderate-to-strong positive correlations between the mean change in PBS scores and BOT-2 Balance component scores for both assessors, as reported in Table 4 and Figure 2, and Table 5 and Figure 3. These findings confirm concurrent validity between the BOT-2 Balance and PBS as measures of functional balance change in spastic CP children, lending support to the use of PBS as a meaningful external anchor for MCID estimation [6,17,25].

**Table 4:** Correlation between mean change of the BOT-2 and PBS score for Assessor 1 ** Correlation is significant at the 0.01 level (2-tailed). Pearson correlation analysis was performed to assess the relationship between change scores. r = correlation coefficient, PBS = Pediatric Balance Scale, BOT-2 = Bruininks–Oseretsky Test of Motor Proficiency Second Edition, A1 = Assessor 1, N = Total number of patients

Pearson Correlations			
Parameters		Mean Difference BOT A1	Mean Difference PBS A1
Mean Difference BOT A1	r-value	1	.604**
	p-value	-	.000
	N	30	30
Mean Difference PBS A1	r-value	.604**	1
	p-value	.000	-
	N	30	30

**Table 5:** Correlation between the mean change of the BOT-2 and PBS score for Assessor 2 ** Correlation is significant at the 0.01 level (two-tailed). Pearson correlation analysis was performed to assess the relationship between change scores. r = correlation coefficient, PBS = Pediatric Balance Scale, BOT-2 = Bruininks–Oseretsky Test of Motor Proficiency Second Edition, A2 = Assessor 2, N = Total number of patients

Pearson Correlations			
Parameters		Mean Difference BOT A2	Mean Difference PBS A2
Mean Difference BOT A2	r-value	1	.583**
	p-value	-	.001
	N	30	30
Mean Difference PBS A2	r-value	.583**	1
	p-value	.001	-
	N	30	30

**Figure 4:**
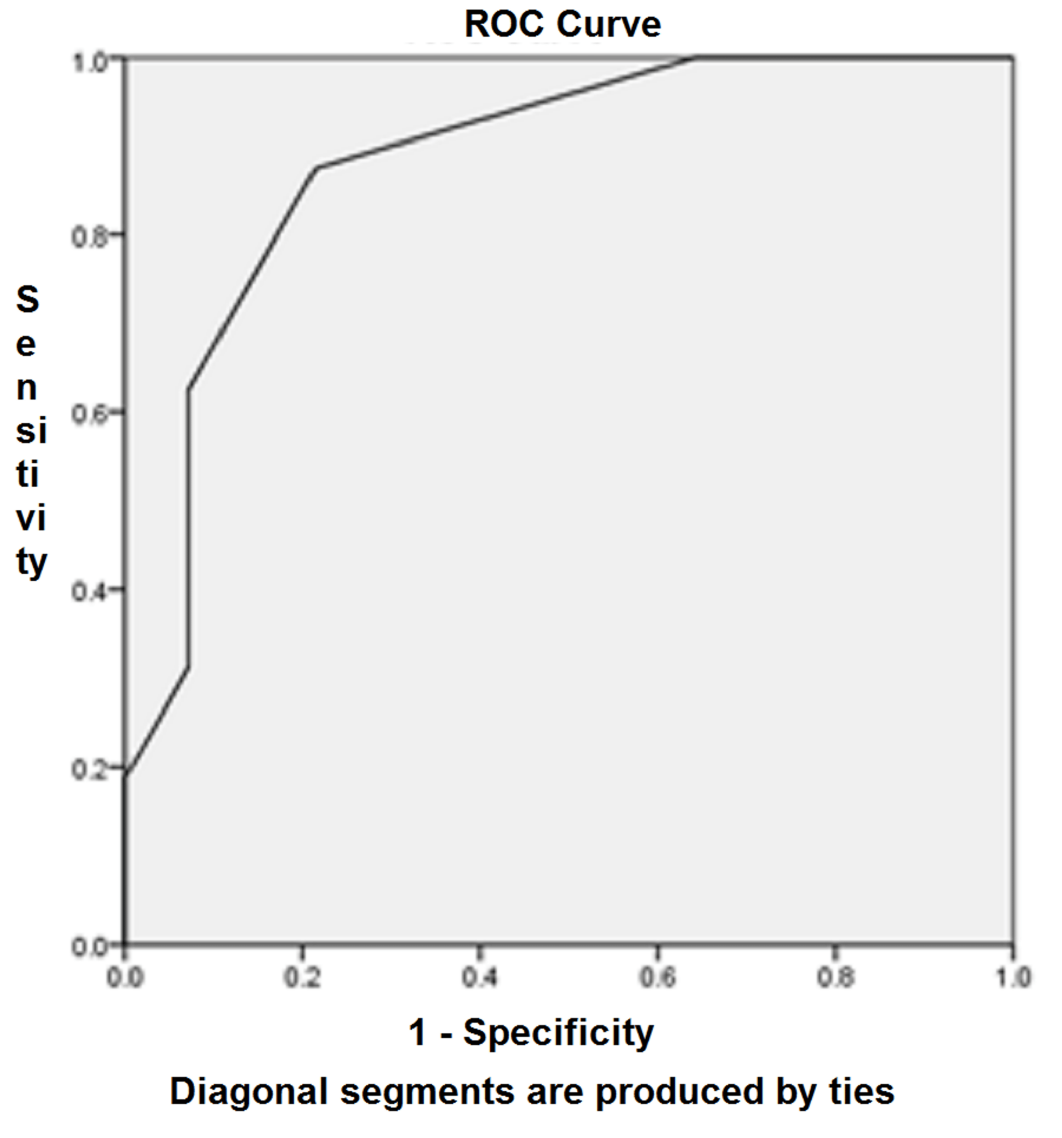
Receiver operating characteristic curve for determining the MCID demonstrating the discriminative ability of the change in BOT-2 Balance subtest scores to identify clinically important improvement using the change in PBS as the external anchor for Assessor 1. Area under the curve = 0.884 MCID = Minimal Clinically Important Difference, BOT-2 = Bruininks–Oseretsky Test of Motor Proficiency Second Edition, PBS = Pediatric Balance Scale The figure was created using IBM SPSS Statistics for Windows, Version 23 (Released 2015; IBM Corp., Armonk, New York).

The MCID was estimated using anchor-based ROC curve analysis with the PBS as the external criterion. For Assessor 1, as reported in Table 6 and Figure 4, the ROC analysis yielded an AUC of 0.884, indicating excellent discriminative ability. The ROC curve coordinates, as reported in Table 7, identified a cut-off of ≥3.5 points as the optimal threshold, achieving a sensitivity of 87.5% and a specificity of 78.6%.

**Table 6:** Area under the curve (MCID analysis with PBS as anchor for Assessor 1) ^a ^Standard error ^b ^Asymptotic significance PBS = Pediatric Balance Scale, BOT-2 = Bruininks–Oseretsky Test of Motor Proficiency Second Edition, A1 = Assessor 1, MCID = Minimal Clinically Important Difference

Test Result Variable(s): Mean Difference BOT A1				
Area	Standard Error^a^	Asymptotic Significance^b^	Asymptotic 95% Confidence Interval	
			Lower Bound	Upper Bound
.884	.06	.00	.75	1.0

**Table 7:** Coordinates of the curve (MCID analysis with PBS as anchor for Assessor 1) ^a ^Standard error PBS = Pediatric Balance Scale, BOT-2 = Bruininks–Oseretsky Test of Motor Proficiency Second Edition, A1 = Assessor 1, MCID = Minimal Clinically Important Difference

Test Result Variable(s): Mean Difference BOT A1		
Positive if Greater Than or Equal To^a^	Sensitivity	1 - Specificity
.00	1.0	1.0
1.50	1.0	.85
2.50	1.0	.64
3.50	.87	.21
4.50	.62	.07
5.50	.31	.07
6.50	.18	.00
9.50	.06	.00
13.0	.00	.00

**Figure 5:**
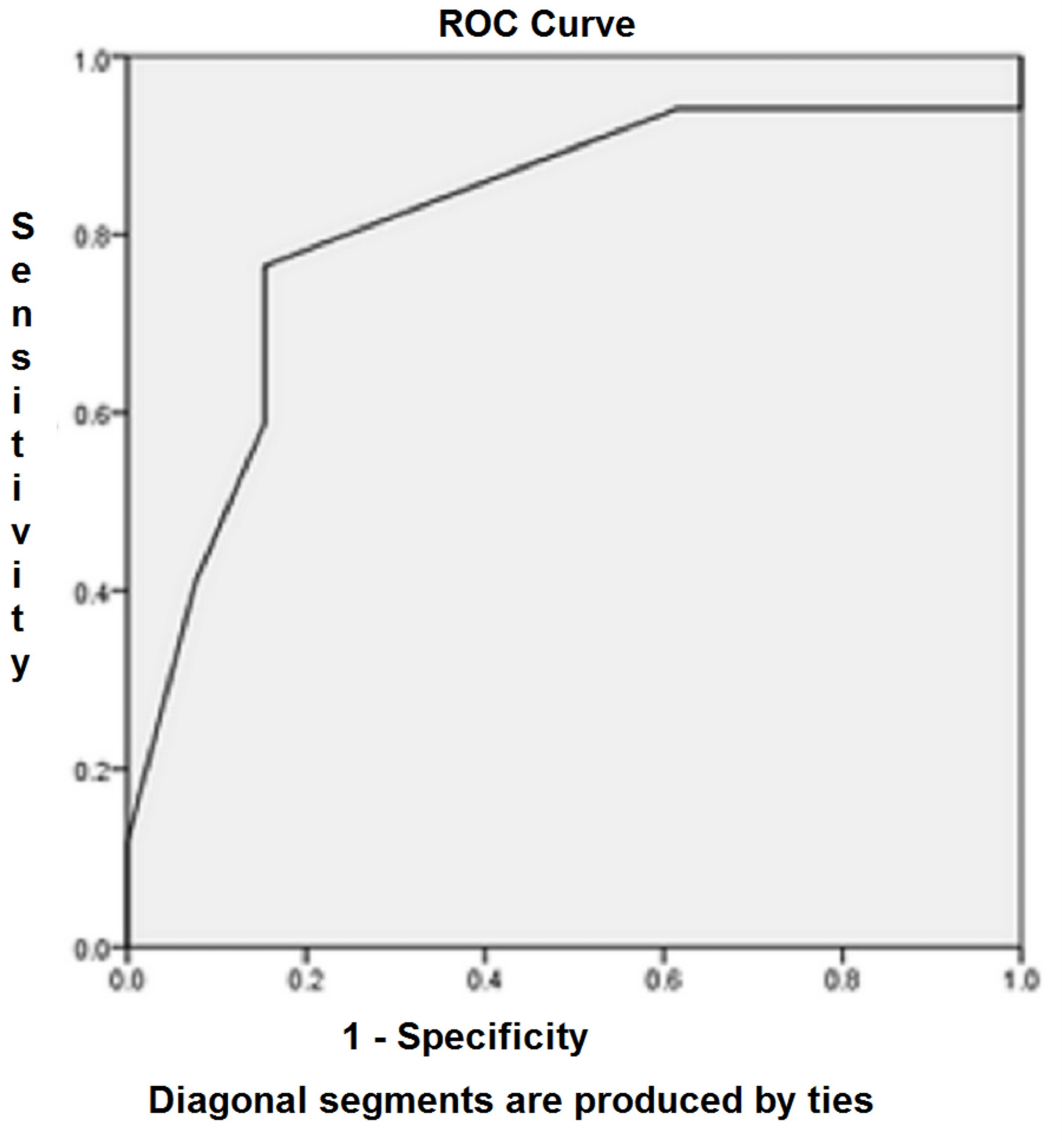
Receiver operating characteristic curve for determining the MCID demonstrating the discriminative ability of the change in BOT-2 Balance subtest scores to identify clinically important improvement using the change in PBS as the external anchor for Assessor 2. Area under the curve = 0.814 MCID = Minimal Clinically Important Difference, BOT-2 = Bruininks–Oseretsky Test of Motor Proficiency Second Edition, PBS = Pediatric Balance Scale The figure was created using IBM SPSS Statistics for Windows, Version 23 (Released 2015; IBM Corp., Armonk, New York).

For Assessor 2, as reported in Table 8 and Figure 5, the AUC was 0.814, reflecting good discriminative capacity. The corresponding ROC coordinates, as reported in Table 9, similarly identified a cut-off of ≥3.5 points, with a specificity of 84.6% and a sensitivity of 76.5%. The convergence of the MCID threshold at ≥3.5 BOT-2 Balance points across both assessors provides robust and clinically meaningful evidence for this value as the MCID for this population [7,22,24,30].

**Table 8:** Area under the curve (MCID analysis with PBS as anchor for Assessor 2) ^a^ Standard error ^b^ Asymptotic significance PBS = Pediatric Balance Scale, BOT-2 = Bruininks–Oseretsky Test of Motor Proficiency Second Edition, A2 = Assessor 2, MCID = Minimal Clinically Important Difference

Test Result Variable(s): Mean Difference BOT A2				
Area	Standard Error^a^	Asymptotic Significance^b^	Asymptotic 95% Confidence Interval	
			Lower Bound	Upper Bound
.814	.083	.004	.65	.97

**Table 9:** Coordinates of the curve (MCID analysis with PBS as anchor for Assessor 2) ^a^ Standard error PBS = Pediatric Balance Scale, BOT-2 = Bruininks–Oseretsky Test of Motor Proficiency Second Edition, A2 = Assessor 2, MCID = Minimal Clinically Important Difference

Test Result Variable(s): Mean Difference BOT A2		
Positive if Greater Than or Equal To^a^	Sensitivity	1 - Specificity
-2.0	1.0	1.0
-.5	.94	1.0
.5	.94	.84
1.5	.94	.69
2.5	.94	.61
3.5	.76	.15
4.5	.58	.15
5.5	.41	.07
6.5	.11	.00
7.0	.05	.00
9.0	.00	.00

**Figure 6:**
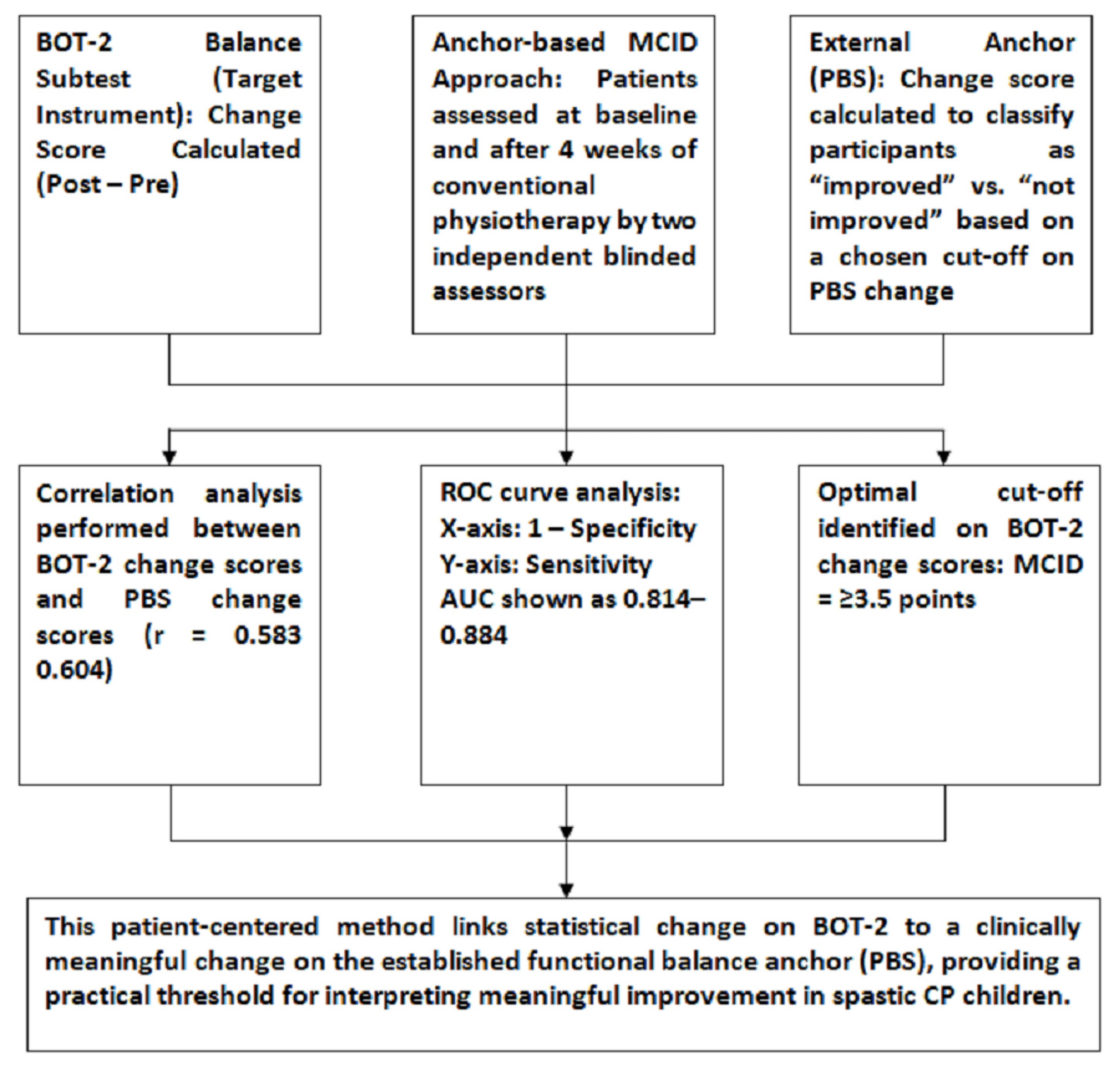
Schematic illustration of the anchor-based approach for determining the MCID of the BOT-2 Balance subtest using the PBS as the external anchor BOT-2 = Bruininks–Oseretsky Test of Motor Proficiency Second Edition; PBS = Pediatric Balance Scale; MCID = Minimal Clinically Important Difference; ROC = Receiver Operating Characteristic; AUC = Area Under the Curve The figure was created using Microsoft Word (Microsoft Corporation, Redmond, WA, USA).

In summary, both the balance subtest of the BOT-2 and PBS scores demonstrate statistically significant and clinically meaningful improvements following four weeks of conventional physiotherapy. The MDC for the BOT-2 Balance component ranged from 1.5 to 1.7 points across assessors, while the MCID was consistently identified at ≥3.5 points using anchor-based ROC analysis, with AUC values of 0.814-0.884, indicating good-to-excellent discriminative capacity. Moderate-to-strong correlations between BOT-2 Balance and PBS change scores further support the concurrent validity of both instruments as responsive balance outcome measures in children with spastic CP [2,6,20,22]. The schematic presentation is demonstrated in Figure 6.

## Discussion

The findings offer important insights into the responsiveness and clinical utility of BOT-2 Balance as an outcome measure in paediatric physiotherapy and address a notable gap in the clinimetric literature pertaining to this population [2,21]. In CP, the most functionally disabling features are deficits in balance (static and dynamic postural control) leading to activity limitation and participation restriction in everyday tasks (walking, playing, and engaging in school-based activities), increasing the risk of falls, and relying on compensatory movement strategies that are biomechanically inefficient, adversely affecting overall quality of life [9-11]. Therefore, standardised and accurate balance measurement, not only for clinical monitoring but also for designing and evaluating targeted interventions, is essential. The balance subtest of the BOT-2 provides a systematic framework, with its structured, norm-referenced tasks that challenge a range of postural demands, for quantifying deficits and tracking improvements over time [2,13,14].

For guiding clinical decision-making, conventional outcome reporting that relies solely on mean differences or statistical significance may be insufficient, as improvements that are perceptible or meaningful to patients, caregivers, or clinicians do not necessarily reflect statistically significant changes. By anchoring the interpretation of change scores in measurement reliability and clinical relevance, respectively, MDC and MCID address this limitation. To confirm that the improvement reflects a genuine shift rather than random fluctuations, the MDC ensures that the observed change exceeds the instrument's inherent measurement error [22]. The MCID provides a threshold that distinguishes truly meaningful gains from statistically trivial ones by identifying the smallest change perceived as clinically beneficial [31].

Across both assessors, the MDC values ranged from 1.5 to 1.7 points for the balance subtest of BOT-2, confirming that the observed mean improvements substantially exceeded measurement error. Furthermore, across both assessors, anchor-based ROC analysis consistently identified an MCID of ≥3.5 points, providing clinicians with an evidence-based, practical benchmark. In spastic CP children, this dual framework of MDC and MCID strengthens confidence in the BOT-2 Balance component as a responsive and interpretable measure.

MDC values for the BOT-2 Balance subtest (1.5-1.7 points) are lower than those reported by Kim et al., who found an MDC_95_ of 9.61 points in children with CP using the balance subtest of BOT-2 [32]. This difference may be attributed to variations in sample characteristics, such as our focus on higher-functioning children (predominantly GMFCS Level I) and different calculation methods. Nevertheless, the current findings align with smaller MDC values reported for other BOT-2 subtests in paediatric populations with mild motor impairments [33].

The MCID of ≥3.5 points identified in our study is consistent with the range of MCID values reported for the PBS in children with CP. Chen et al. reported MCID values ranging from 3.66 to 5.83 points for the PBS total score in children with CP, indicating that our BOT-2 MCID threshold reflects a comparable magnitude of clinically meaningful change in functional balance [30]. These comparisons support the responsiveness of the BOT-2 Balance component as a useful complementary tool to the PBS in paediatric rehabilitation [34]. This convergence of findings across independent studies reinforces the clinical utility of establishing population-specific MDC and MCID benchmarks for balance assessment tools in spastic CP.

Between changes in the PBS and BOT-2 Balance component scores, moderate-to-strong Pearson correlations provided meaningful evidence of concurrent validity [9,15-17]. These correlations suggest that while both tools assess balance-related constructs, they do not measure identical domains. The BOT-2 Balance component evaluates postural control within a broader, standardised motor proficiency framework, while PBS focuses primarily on functional balance tasks relevant to activities of daily living. In clinical practice, the complementary nature of these two tools implies that their combined use may offer a more comprehensive and multidimensional picture of the balance capabilities of a child than either instrument alone [25,26,35]. In paediatric rehabilitation for informed treatment planning, this is particularly valuable where accurate and appropriate assessment is essential.

In studies involving typically developing children and those with developmental coordination disorder, autism spectrum disorder, and intellectual disabilities, the BOT-2 is validated [5,15-17]. However, in children with CP, the evidence has remained limited. In CP, excellent reliability of the BOT-2 was reported by Selves et al. [6], but the study did not establish MCID thresholds, representing a significant gap, which is addressed by the current study. In spastic CP, by providing the first evidence-based MCID value for the BOT-2 Balance component, the findings of the current study extend this literature, thereby enabling clinicians to interpret change scores with greater precision and confidence.

From comparable paediatric balance tools, the identified MCID of ≥3.5 points is consistent with clinimetric findings. In school-aged CP children, the sensitivity of the PBS to clinical change was demonstrated by Franjoine et al. [20], and in spastic CP, the validity was confirmed by Yi et al. [35]. Across both independent assessors, the generalisability within the study population is reported by the convergence of optimal cut-off values at ≥3.5 points supported by AUC values of 0.814-0.884, which further reinforces the appropriateness of this threshold.

Across all outcome measures, the use of two trained independent assessors permitted evaluation of inter-rater consistency and minimised observer bias. By employing the PBS as an external clinical criterion, the anchor-based ROC curve approach is methodologically rigorous and yields MCID thresholds with demonstrable discriminative capacity [7,24]. The internal validity of the results is further strengthened through cross-validation of improvements across both balance subtests of the BOT-2 and PBS. Additionally, adherence to institutional ethical approval and the acquisition of written informed consent from all parents and guardians ensured that the study was conducted in accordance with established research ethics standards.

Clinically, to distinguish genuine therapeutic gains from measurement in routine assessments, the establishment of an MDC of 1.5-1.7 points and an MCID of ≥3.5 points for the BOT-2 Balance component equips physiotherapists with objective benchmarks. When a spastic CP child achieves an improvement of ≥3.5 points following an intervention on the balance subtest of the BOT-2, clinicians may be confident that this change is both clinically meaningful and statistically reliable [28,36,37].

The concurrent validity reported with the PBS supports the use of the balance subtest of BOT-2 as a complementary outcome measure, enabling a more comprehensive evaluation of motor proficiency alongside functional balance. In practice, this allows therapists to set realistic, evidence-informed goals, facilitating improved treatment planning by distinguishing true improvement from measurement error, leading to evaluation of more precise outcomes; through the use of clear, interpretable thresholds enhancing communication with families and caregivers; and more defensible resource allocation, as for the continuation or modification of therapy, clinically meaningful improvements provide stronger justification [6,17,25,26].

From a broader perspective, the availability of MCID data for BOT-2 Balance strengthens the foundation for evidence-based practice in paediatric rehabilitation. It supports funding and policy advocacy by enabling the demonstration of clinically significant outcomes and provides a valuable teaching resource for physiotherapy education. Furthermore, given the global prevalence of CP, standardised clinimetric data for widely used tools such as BOT-2 facilitate cross-cultural research collaboration and international comparison of intervention outcomes [1,3,29]. The integration of BOT-2 Balance into routine clinical pathways, encompassing baseline assessment, goal setting, intervention monitoring, and outcome evaluation, is therefore both feasible and strongly recommended based on the evidence presented in this study.

Limitations

First, with respect to sample characteristics, the overwhelming majority of participants (93.3%) were classified at GMFCS Level I, representing a relatively mild severity profile. This limits the generalisability of the MDC and MCID values to children with GMFCS Levels III-V, who constitute a substantial and clinically important segment of the CP population. Second, convenience sampling was employed due to practical constraints. Although all eligible children presenting to the participating centres during the study period were approached consecutively, this method may have introduced selection bias by over-representing families with better access to tertiary care services and higher health literacy. Third, the final sample size was substantially smaller because it was a pilot study, and the formal sample size calculation was not hypothesis-driven. Fourth, intervention variability represents a notable limitation, as the type, intensity, frequency, and duration of physiotherapy received by individual children across different treatment centres were not standardised or controlled; however, this design allowed us to assess the responsiveness of the BOT-2 Balance subtest in detecting both large and small changes under real-world conditions. Future studies should consider standardised intervention protocols to better control for these variables. Fifth, evaluation of the long-term responsiveness of the BOT-2 Balance component or the durability of clinical improvements beyond the observation period was not performed, which precludes strong causal inference.

Recommendations for future research

Future studies should include children across all GMFCS levels and across the full spectrum of CP subtypes to establish MCID thresholds that are generalisable to the broader CP population. Longitudinal studies over extended periods would provide stronger evidence regarding the sustained responsiveness of the tool and the durability of clinically meaningful change. Future research should also explore functional outcome linkages, examining whether improvements in balance scores of BOT-2 correspond to measurable gains in real-world activities such as gait quality, participation, and quality of life, thereby enhancing the ecological validity of the measure. Finally, the development of age- and severity-stratified normative data for the BOT-2 Balance subtest in CP would substantially enhance its clinical interpretability and support more individualised goal setting and outcome evaluation.

## Conclusions

The present pilot study provides preliminary evidence-based MDC and MCID values for the BOT-2 Balance component in spastic CP children. The MDC points confirm that the improvements observed in BOT-2 Balance scores following four weeks of conventional physiotherapy genuinely exceeded the measurement error for both assessors. The MCID, across both independent assessors, constitutes a clinically meaningful and statistically robust threshold for interpreting balance improvement in this population. The moderate-to-strong correlations between PBS change scores and BOT-2 Balance component further substantiate the concurrent validity of the BOT-2 Balance component as a responsive outcome measure and support its complementary use alongside the PBS for comprehensive balance evaluation. Statistically significant pre- to post-intervention improvements were observed across all BOT-2 and PBS assessments, reinforcing the sensitivity of both instruments to clinically relevant change. However, given the small sample size and restriction to higher-functioning children, these findings should be interpreted with caution and considered preliminary. Future larger-scale studies including children across the full spectrum of GMFCS levels are needed to validate and generalise these clinimetric benchmarks. For spastic CP children, these findings support the integration of the balance component of BOT-2 into routine clinical assessment pathways, enabling clinicians to set meaningful rehabilitation goals, evaluate intervention effectiveness with greater precision, and communicate outcomes more transparently to families and stakeholders.
